# ZnO-nanostructure-based electrochemical sensor: Effect of nanostructure morphology on the sensing of heavy metal ions

**DOI:** 10.3762/bjnano.9.227

**Published:** 2018-09-11

**Authors:** Marina Krasovska, Vjaceslavs Gerbreders, Irena Mihailova, Andrejs Ogurcovs, Eriks Sledevskis, Andrejs Gerbreders, Pavels Sarajevs

**Affiliations:** 1 G. Liberts' Innovative Microscopy Centre, Institute of Life Sciences and Technology, Daugavpils University, Daugavpils LV-5401, Latvia

**Keywords:** cadmium, electrochemical sensors, heavy metal ions, lead, one-dimensional nanostructures, ZnO

## Abstract

ZnO nanostructures are promising candidates for use in sensors, especially in electrochemical sensors and biosensors, due to their unique physical and chemical properties, as well as sensitivity and selectivity to several types of contamination, including heavy metal ions. In this work, using the hydrothermal method, nanostructures of ZnO were synthesized in four different morphologies: nanorods, nanoneedles, nanotubes and nanoplates. To determine the peculiarities of adsorption for each morphology, a series of electrochemical measurements were carried out using these nanostructured ZnO coatings on the working electrodes, using aqueous solutions of Pb(NO_3_)_2_ and Cd(NO_3_)_2_ as analytes with different concentrations. It was found that the sensitivity of the resulting electrochemical sensors depends on the morphology of the ZnO nanostructures: the best results were achieved in the case of porous nanostructures (nanotubes and nanoplates), whereas the lowest sensitivity corresponded to ZnO nanorods with a large diameter (i.e., low surface-to-volume ratio). The efficiency of sedimentation is also related to the electronegativity of adsorbate: it has been shown that all observed ZnO morphologies exhibited significantly higher sensitivity in detecting lead ions compared to cadmium ions.

## Introduction

Today, due to the rapid growth of industry and transport, the issues of environmental pollution by heavy metals, particularly by lead, mercury, cadmium and cobalt, are becoming more and more topical. Many metals form stable organic compounds that dissolve well in water and result in the migration of heavy metal ions in aquatic and terrestrial systems, thereby resulting in high levels of contamination [1–2].

Lead is one of the most harmful heavy metals. Its presence is hazardous to the environment, it is toxic and carcinogenic, and it tends to accumulate in living organisms, where it replaces other elements in bones and tissues, and causes long-term poisoning [3–5]. That is why the creation of a sensor that could detect the presence of lead and other heavy ions, even in small quantities, is an important and topical task.

ZnO nanostructures are promising candidates for use in such sensors. They are sensitive to various types of contamination, including almost all heavy metal ions and organic pollutants, and show very good adsorption results for the most hazardous ions (Pb, Cd, Hg) [6–8]. Generally, ZnO is chemically inactive, so it will not become a secondary source of contamination. Furthermore, ZnO nanostructures can have various types of morphologies, thus making it possible to diversify the electrical and optical properties of the nanostructured layer for the particular application without changing the actual substance. Contrary to the case of needle-shaped nanostructures, other types of ZnO nanostructures have been less investigated, whereby research on such structures could open the possibilities for more potential applications.

Electrochemical methods such as cyclic voltammetry (CV), impedance spectroscopy (IS) and differential pulse voltammetry (DPV) are highly efficient in both qualitative and quantitative analysis of solutions [9–11]. These methods allow for the detection of hazardous chemicals even at very low concentrations [12–14]. Furthermore, combined with a sensor platform based on nanomaterials and nanostructures, these electrochemical methods contribute to the improvement of sensor performance in terms of sensitivity and specificity, primarily as a result of the large working electrode surface area, and consequently, large number of active links that raises the overall sorption capability.

Our research team performed a series of experiments aimed at determining the influence of hydrothermal growth conditions on the parameters of the obtained nanostructures, as well as obtaining ZnO nanostructures in various morphologies, such as ZnO nanoneedles, nanorods, nanotubes, nanoplates, etc. Also the purpose of our previous research was to identify optimal growth parameters for obtaining a homogeneous, dense, well-aligned nanostructured ZnO coating with good adhesion to hard surfaces. The results of the experiments are displayed and summarized in previously published articles [15–18].

This article is a follow-up study of previous research and describes the peculiarities of the practical application of previously obtained ZnO morphologies as a working electrode for the electrochemical sensor of heavy metal ions.

In this work, using the hydrothermal method, nanostructures of ZnO were synthesized in four different morphologies: nanorods (NRs), nanoneedles (NNs), nanotubes (NTs) and nanoplates (NPs). The samples described in this article were obtained using a preparation protocol based on previously recognized optimal hydrothermal growth parameters and are characterized by increased homogeneity, density, and orderliness in comparison with those obtained in early studies. To determine the peculiarities of adsorption for each morphology, a series of electrochemical measurements were carried out using these nanostructured ZnO coatings on the working electrodes using aqueous solutions of Pb(NO_3_)_2_ and Cd(NO_3_)_2_ as analytes with different concentrations.

## Experimental

### Design and fabrication of the ZnO nanostructure-based electrochemical sensor

Three morphologies of well-aligned one-dimensional ZnO nanostructures were selected for the experiment: nanorods, nanoneedles and nanotubes. The samples were prepared according to the following description.

Glass slides (76 × 26 × 1 mm) were precleaned via ultrasonication in aqueous solutions of KOH and H_2_SO_4_, followed by several rinses with deionized and distilled water, and finally dried in an electric oven at 90 °C to remove any residual water.

**Figure 1:**
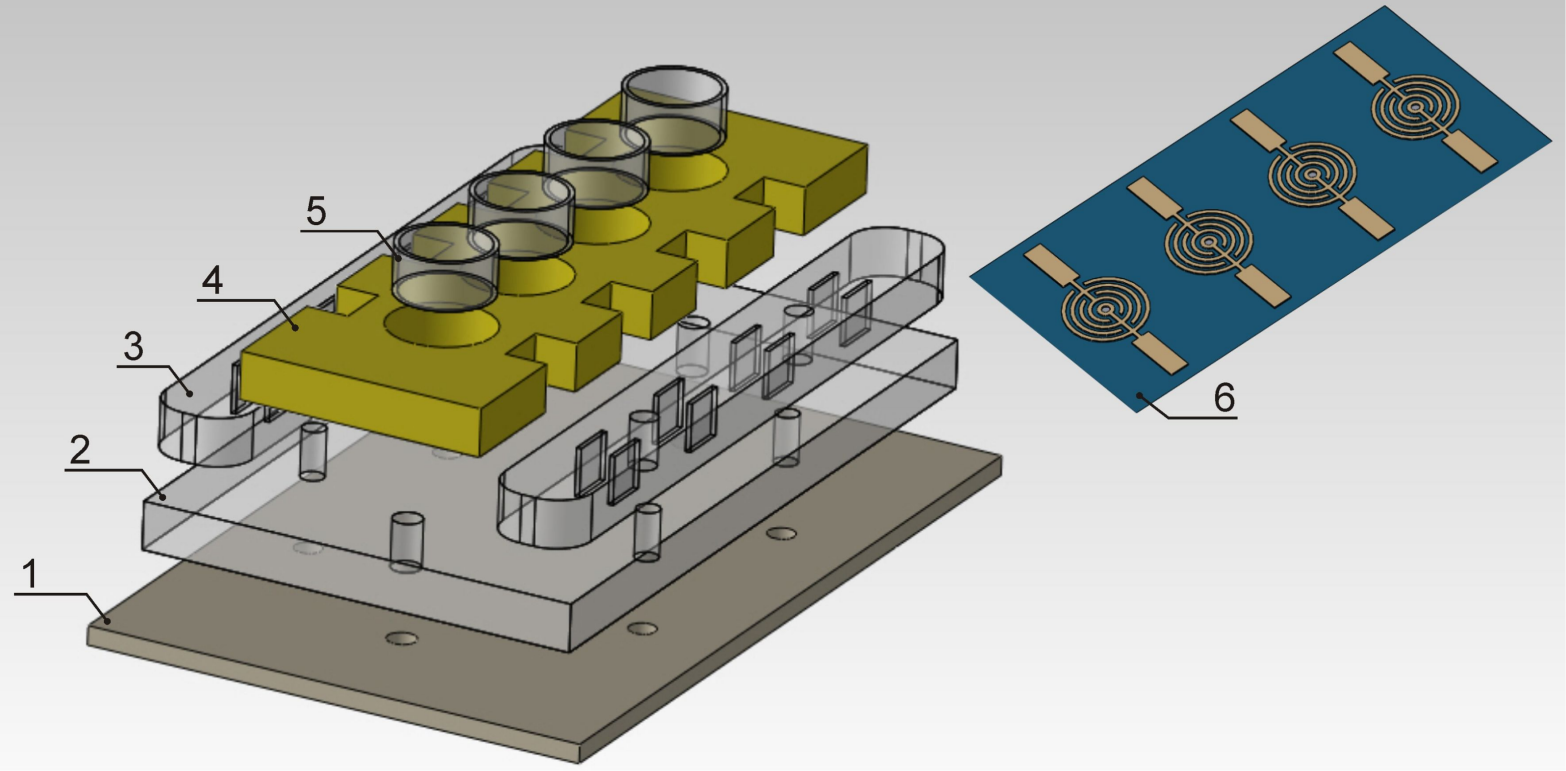
Structure diagrams of the electrical measurement cell and electrodes. Measurement cell consists of the following parts: a corps (1), an interlayer (2) with push-in contacts (3) and a sealing rubber mask (4) with replaceable plastic cylinders (5). The complete sample (6) consists of four electrodes, which allow four independent measurements to be consistently performed for solutions of different concentration or chemical composition.

First, a 100 nm thick chrome layer was sputtered onto the glass substrate through a metal shadow-mask to provide electrical conductivity for further electrochemical measurements. The deposition was carried out with the LAB18 thin film deposition system (Kurt J. Lesker, USA) in DC magnetron sputtering mode. As a result, a set of electrodes consisted of four mutually separated planar elements, as illustrated in Figure 1.

All of the manipulations described below were carried out using a metal mask, which limits the flow of liquid around the sample and reduces the probability of grown particles to enter covered areas, providing the necessary selectivity of nanostructure growth only on the electrodes.

In the second step a temporary extra rubber sealing mask was applied and Cr electrodes were coated with a ZnO seed layer in order to provide adhesion of the nanostructured ZnO film to the glass surface, as well as epitaxial growth and the vertical alignment of nanostructures. It was prepared by dip coating using 5 mM zinc acetate (Zn(O_2_CCH_3_)_2_) solution in ethanol. The seed layer film was dried in a stream of dry nitrogen and thermally treated at 350 °C for 30 min in air, such that zinc acetate was completely converted into zinc oxide. Subsequently, the samples were slowly cooled to room temperature.

The nanostructured layer of the electrode was obtained by the hydrothermal synthesis method using an aqueous solution of zinc nitrate hexahydrate (Zn(NO_3_)_2_·6H_2_O; 99% purity) and hexamethylenetetramine (HMTA; C_6_H_12_N_4_; 99% purity), where zinc nitrate is used as a source of Zn^+^ ions; however, HMTA acts as a pH buffer by slowly releasing OH^−^ ions. The decomposition rate of HMTA increases with the temperature of the growth solution. The growth of ZnO nanostructures occurs between 80–90 °C, where the temperature is sufficient to produce the required amount of OH^−^ ions from the HMTA solution in a relatively short period of time. In addition, the reaction does not occur instantaneously and not all of the components fall out as sediments. Furthermore, maintaining the temperature below the boiling point prevents any intense evaporation of the working solution, thereby allowing the experiment to be carried out without the use of an autoclave.

In order to grow uniform and vertically aligned ZnO nanorod arrays, the ZnO seed-coated glass substrate was immersed in an aqueous solution of 0.1 M Zn(NO_3_)_2_ + 0.1 M C_6_H_12_N_4_ in a glass vessel. The vessel was sealed with a lid and placed in oven maintained at 90 °C for three hours. Afterwards, the glass vessel was removed from the oven and cooled to room temperature. The sample was then removed from the solution, rinsed several times with distilled water and thermally treated at 90–110 °C for 30 min in order to remove the adsorbed water. For more detailed information see [17–18].

ZnO nanotube arrays were obtained using a self-selective etching method based on selective dissolution of metastable ZnO planes with decreasing growth solution temperature, achieving a considerable Zn-ion deficiency during the hydrothermal process [19–22]. The synthesis of the ZnO nanotubes was performed in a growth solution with the same chemical composition as for synthesis of nanorods: equimolar aqueous solution of 0.1 M Zn(NO_3_)_2_ + 0.1 M C_6_H_12_N_4_. The growth process was divided into two stages: growth of ZnO nanorods at 90 °C for three hours, and selective etching of ZnO nanorods at 50 °C for eighteen hours. After the growth process, the samples were rinsed several times and dried. More detailed information can be found in [15–16].

The ZnO nanoneedles were synthesized by increasing the solution’s pH level to form nanorod arrays with reduced diameters and higher aspect ratios. In order to maintain the chemical purity of the samples, the same chemical components were used for synthesis. The increase in pH was provided by not using an equimolar ratio of components, as in the previous cases, but instead, a 1:4 ratio with excess HMTA. An aqueous solution of 0.025 M Zn(NO_3_)_2_ + 0.1 M C_6_H_12_N_4_ was used to allow an increase in the pH of the working solution from 5.6 to 7.6.

In the time interval between the end of the growth process of nanostructures and the performance of electrochemical measurements, the electrodes were stored under ambient conditions.

### Characterization

The surface morphology of the processed samples was investigated using a scanning electron microscope (SEM). The chemical composition of the samples was determined by an Oxford Instruments INCAx-act energy dispersive spectrometer (EDS) X-ray detector attached to the SEM. To determine the structural and phase composition, the XRD spectra were recorded on a SmartLab Cu Kα (λ = 1.543Å) diffractometer (RIGAKU, Japan) with parallel beam geometry using an additional Ge(220)×2 monochromator. The obtained X-ray pattern peaks were analysed by the PDXL program and compared to a database [23].

### Electrochemical studies

The electrochemical measurements were performed on the Versa Stat 3 potentiostat supplemented by a laboratory-developed electrical measurement cell, which is consistent with the geometry of aforementioned samples (See (6) in Figure 1).The schematic diagram of the electrical measurement cell and the sample is shown in Figure 1.

The reference cell consists of a corps (1), an interlayer (2) with push-in contacts (3) and a sealing system, which consists of chemically inert rubber seals (4) with holes of the same size as the electrode diameter and replaceable plastic cylinders (5). The sealing system prevents the solution from leaking, and allows for the application of the required amount of solution directly onto the electrodes. The cells were connected to a set of switches, whereby one can turn each of the four electrodes sequentially on and off. The measurements were performed at 20 °C in the potential range of −2 V to +2 V at a scan rate of 0.5 Vs^−1^.

For a qualitative analysis of the lead-ion deposition process, a series of cyclic voltammetry (CV) measurements were carried out using aqueous Pb(NO_3_)_2_ solution with concentrations of 150 μM, 300 μM, 1.5 mM, and 3 mM as a source of Pb^2+^ ions. In order to determine and compare the sensor sensitivity to other heavy metal ions, the experiment was repeated with cadmium, where the same concentration of aqueous Cd(NO_3_)_2_ solution was used as a source of Cd^2+^ ions. The aqueous solutions of analytes were freshly prepared prior to measurement.

In order to determine the sensor sensitivity threshold and quantify the change in ion concentration, a series of DPV measurements in Pb(NO_3_)_2_ aqueous solution at concentrations of 1.5 μM, 3 μM, 15 μM, 30 μM, 150 μM, and 300 μM were performed. The same configuration of the cell was used in the measurements.

## Results and Discussion

### Characterization of the ZnO nanostructure-modified electrodes

**Figure 2:**
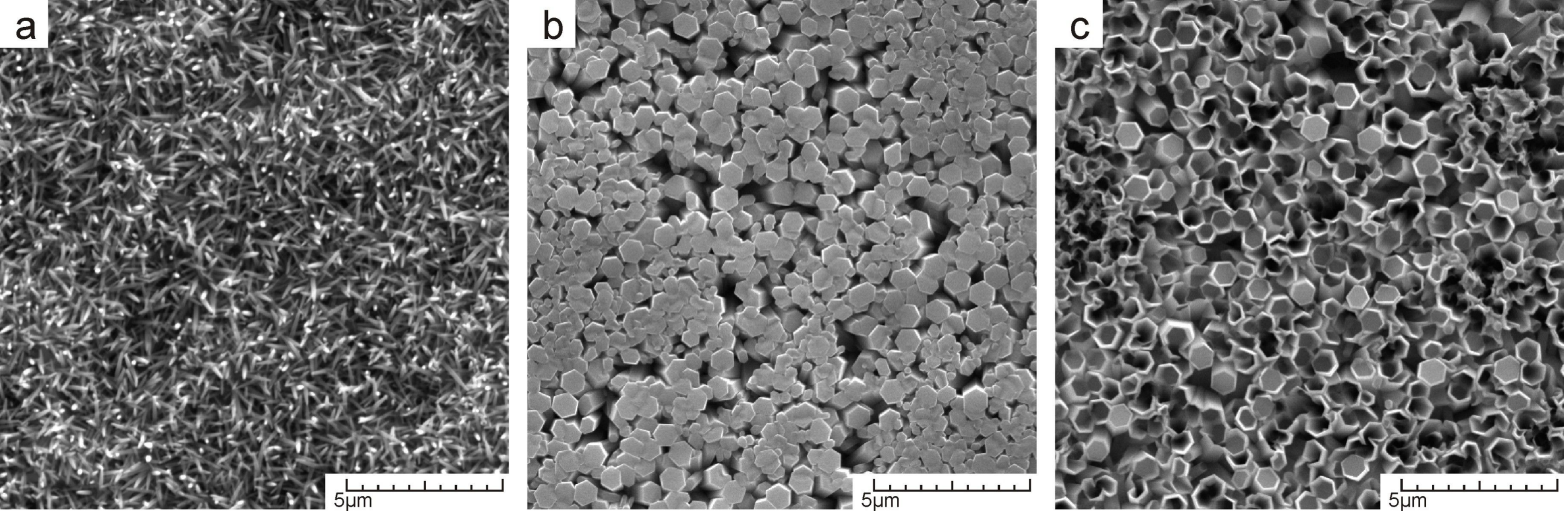
SEM images of as-synthesized ZnO nanostructure arrays: a) nanoneedles, b) nanorods [15], c) nanotubes [15].

SEM images show that the resulting nanostructured coatings of all three morphologies were dense, homogeneous and of well-controlled shapes (see Figure 2).

**Figure 3:**
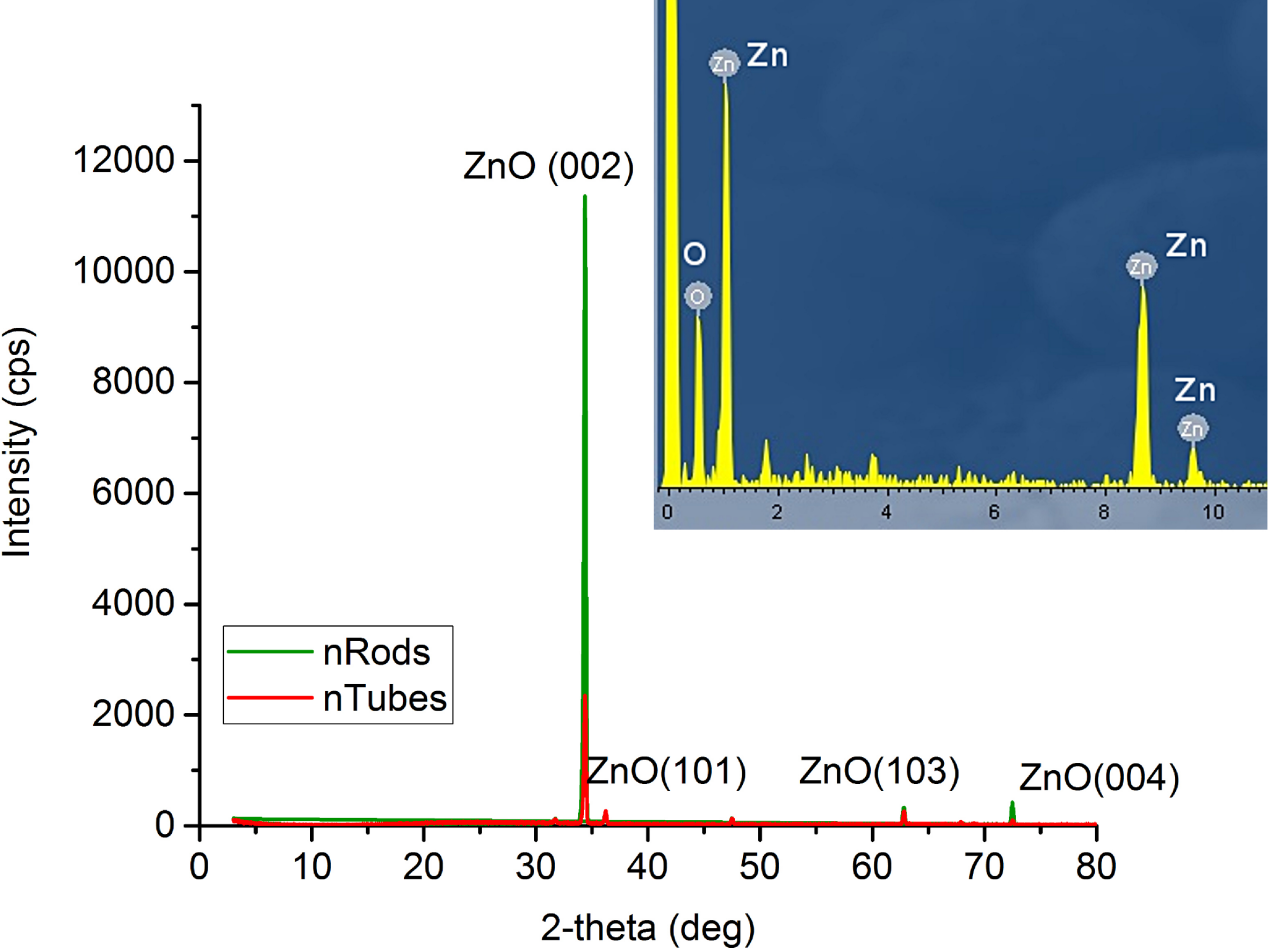
XRD pattern of ZnO nanorod and nanotube arrays. Inset: EDS spectrum of the as-synthesized samples.

The X-ray diffraction results revealed that the samples are crystalline with a hexagonal wurtzite structure. All XRD spectra show one dominant diffraction peak along the (002) plane, whereas the intensity of all the remaining peaks is negligible, confirming that the ZnO nanostructure arrays are well-aligned and have a strong preferential orientation in the (002) plane direction (Figure 3). Low levels of amorphous background reveal that the nanostructures have a high degree of crystallinity. In the case of the nanotubes, the intensity of the peak corresponding to the (002) plane is about four times lower than in the case of nanorods, indicating good efficiency of metastable plane etching.

The EDS spectrum confirms that the as-synthesized samples are chemically pure: no other elements other than Zn and O were detected.

### Cyclic voltammetry studies

Prior to the CV measurements, 250 μL of distilled water was pipetted into each cell, left for ten minutes to ensure the complete wetting of the nanostructured surface, and then the CV curves were recorded. It was determined that the CV curves for all four cells are very nearly identical. This allows for the exclusion of the effect of cell specificity on the subsequent measurement result.

**Figure 4:**
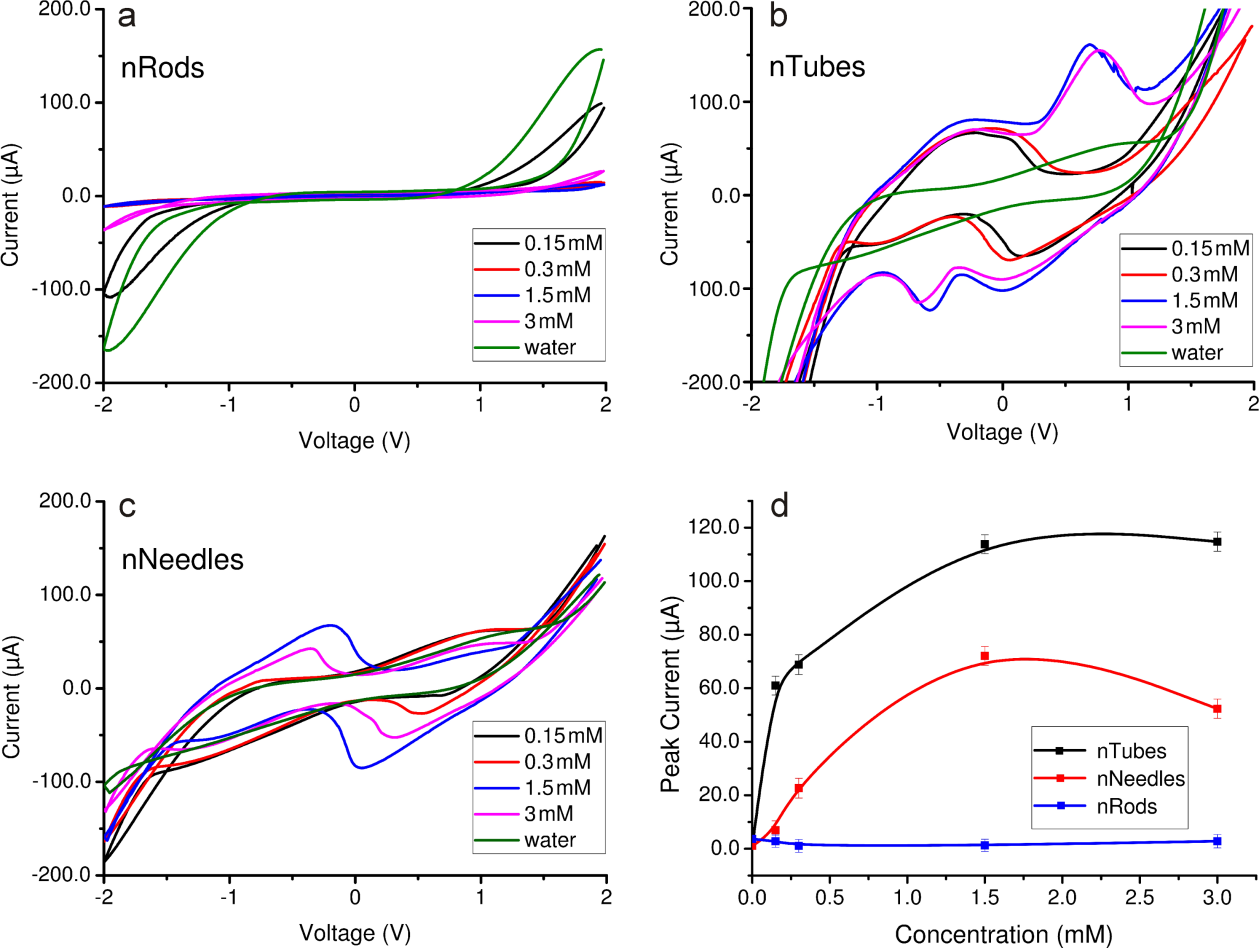
CV curves of aqueous Pb(NO_3_)_2_ solution on ZnO electrodes with different morphologies: (a) nanorods, (b) nanotubes, (c) nanoneedles, and (d) CV reduction peak dependence on Pb(NO_3_)_2_ concentration for all morphologies.

Subsequently, the experiments were conducted by using aqueous Pb(NO_3_)_2_ solutions with concentrations of 150 μM, 300 μM, 1.5 mM, and 3 mM. From the recorded CV curves, it can be seen that, in the case of nanorods, there is no significant difference with increasing concentration of Pb(NO_3_)_2_ – all curves almost overlap (Figure 4a).

In the case of nanotubes there are significant differences in the measured CV characteristics, as outlined in Figure 4b. There is an asymmetry between the upper (oxidation) and the lower (reduction) components of the CV loop, which confirms that the process is irreversible: strong forces of attraction occur between metal ions and the ZnO nanostructure surface, resulting in sedimentation containing a mixture of lead oxides and metallic lead. The XRD pattern and EDS spectra of the sediment composition has been previously reported [16]. The formation of lead oxide is explained by the quasichemical Lewis interaction between lead ions and hydroxy groups on the ZnO surface as a result of coordination or covalent bonding. The presence of metallic lead in the sedimentation can be explained by the electrostatic attraction between Pb^2+^ and ZnO nanotube defects formed in large amount during the etching process.

The quantitative dependence of the concentration is also traceable: the size of the peak correlates with the concentration of the analyte. Duplex sets of CV curves are evident, in which the concentration differs by a factor of two and the duplex concentrations differ from each other by a factor of up to ten. At high concentrations, a second reduction peak occurs and the asymmetry of the CV loop becomes even larger and could be expressed not only in signal intensity, but also in the form of a curve. It confirms that the processes of ion sedimentation are irreversible.

In the case of nanoneedles, the asymmetry between oxidation and reduction components is less obvious (Figure 4c). The peak intensity is much lower than in the case of nanotubes. It may indicate that the bond strength between lead ions and the surface of ZnO is weaker. No proportional ratio of concentrations was observed.

Figure 4d shows the dependence of the reduction peak maximum on the solution concentration, proving that nanotubes are more sensitive toward Pb^2+^.

A significant increase of the sensing properties of ZnO nanotubes in comparison with nanorods is confirmed by the determination of the capacity of static adsorption for Pb ions described in [16]. The higher value of this parameter also indicates an increase in the total number of bonds of lead ions to the surface of ZnO nanotubes. Calculations show that the static adsorption capacity of ZnO nanotubes and nanorods obtained under the same conditions is 611 mg/g and 256 mg/g, respectively. The measurements were taken at room temperature using a 300 mM/L solution and immersing for 5 min.

**Figure 5:**
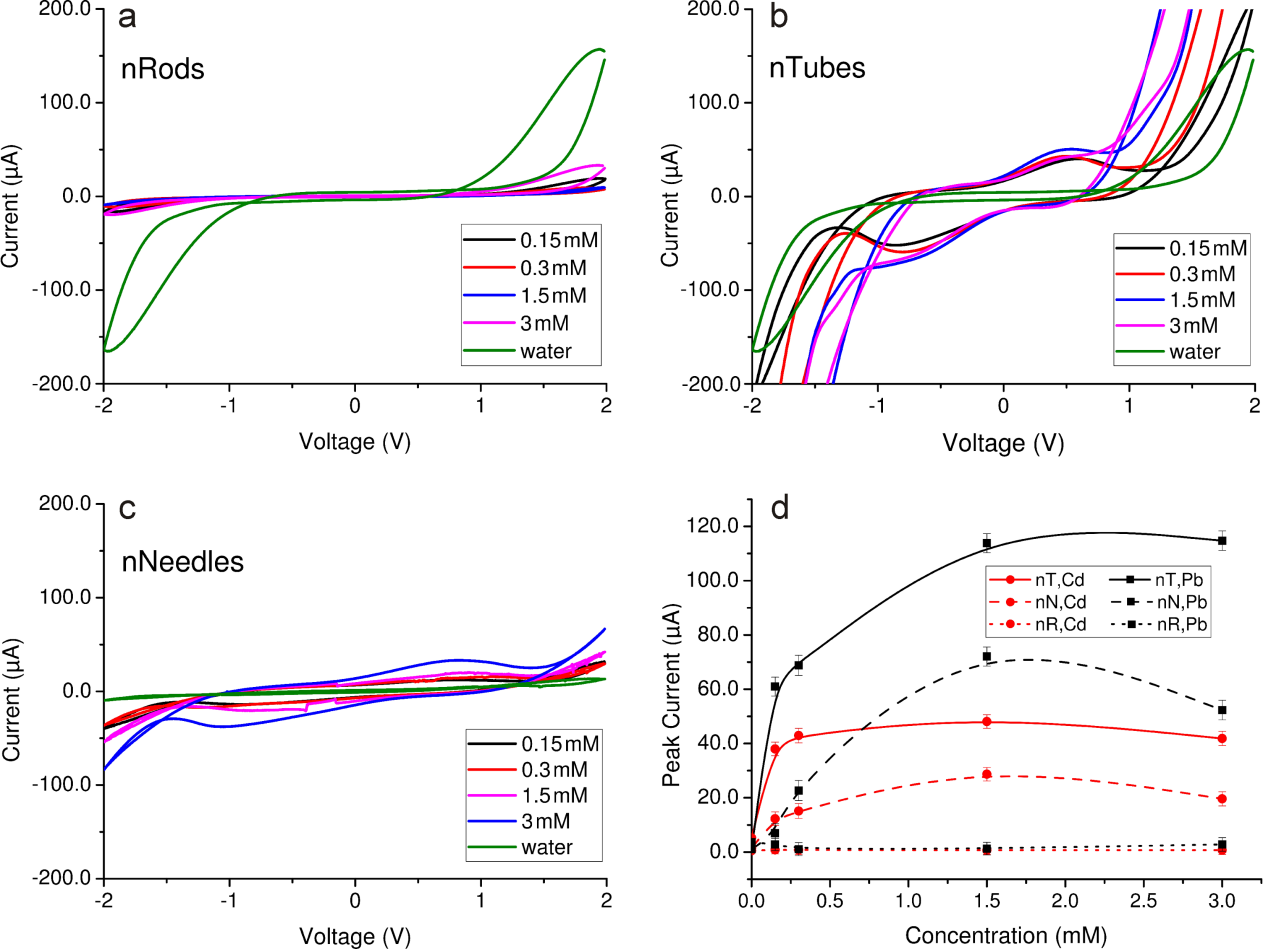
CV curves of aqueous Cd(NO_3_)_2_ solution on ZnO electrodes with different morphologies: (a) nanorods, (b) nanotubes, (c) nanoneedles and (d) CV reduction peak dependence on Cd(NO_3_)_2_ concentration for all morphologies and its comparison with the analogous curves for Pb(NO_3_)_2_ concentration (the data taken from Figure 4d).

To study the adsorption of other ions, the experiment was repeated using the same concentrations of Cd(NO_3_)_2_ aqueous solutions. In general, CV curves for Cd(NO_3_)_2_ solutions had the same tendency as for Pb(NO_3_)_2_ solutions: ZnO nanorods exhibit no sensitivity to changes in solution concentrations (Figure 5). Slightly better results are observed in the case of ZnO nanoneedles, although the difference in concentration is much less noticeable than for the Pb(NO_3_)_2_ solution. ZnO nanotubes show the best results, similar to the case of the Pb(NO_3_)_2_ solution: duplex concentrations are observed, however, the differences are less noticeable than those of Pb(NO_3_)_2_.

If the reduction peaks of these two substances are compared (see Figure 5d), it can be seen that, for morphologies with higher sensitivity to both metal ions (i.e., nanotubes and nanoneedles), the current in the case of Pb(NO_3_)_2_ (black curves) is almost three times higher than that of the Cd(NO_3_)_2_ solution (red curves). This confirms a significantly better adsorption of Pb ions on the surface of ZnO than Cd ions.

The processes of ion sedimentation can be explained as follows. It is well known that the interaction of ZnO with airborne water or water vapour results in the formation of negatively charged hydroxy (OH^−^) groups, which can themselves act as new adsorption sites on the ZnO surface. The adsorption mechanism is based on the interaction of positively charged metallic ions with negatively charged hydroxy groups on the ZnO surface and, consequently, Zn–O–H bonds are replaced by Zn–O–Cd or Zn–O–Pb bonds [24–25]. Evidently, the better results in adsorption of Pb^2+^ compared to Cd^2+^ can be explained by the electronegativity of metals [25]. Since lead has a higher electronegativity than cadmium, it is more likely to be involved in the substitution reaction and forms stronger bonds with the ZnO surface. The Freundlich (multilayered) adsorption model is the result of a very strong interaction [26]. By contrast, Cd–O bonds are much weaker than Pb–O bonds, so in the case of cadmium, the Langmuir (monolayer) adsorption model is valid [6–7,27]. This is evidenced by lower sensor sensitivity to changes in concentrations of Cd(NO_3_)_2_: the saturation occurs by filling all possible free bonds at low concentrations (which leads to the formation of monolayers; as a result, subsequent layers are not formed due to weak bonds.

Figure 5d shows that, in all cases of ZnO morphologies, the current of Cd ions saturated at low concentrations (up to 0.3 mM), and then remained nearly constant for increasing concentrations. Comparatively, in the case of Pb ions, the current saturated at much higher concentration of analyte (in the range of 1.5 to 2 mM). The reduction peak current for the highest concentration can be explained by mass crystallization in the solution, in parallel with the sorption process. It is the threshold above which crystallites of lead oxides become visible on the ZnO nanostructured surface by SEM.

In order to visually compare the sedimentation characteristics of both substances, the concentration of nitrate was increased 100 times (to 300 mM), which led to an intense crystallization process and the formation of visually appealing sediments.

**Figure 6:**
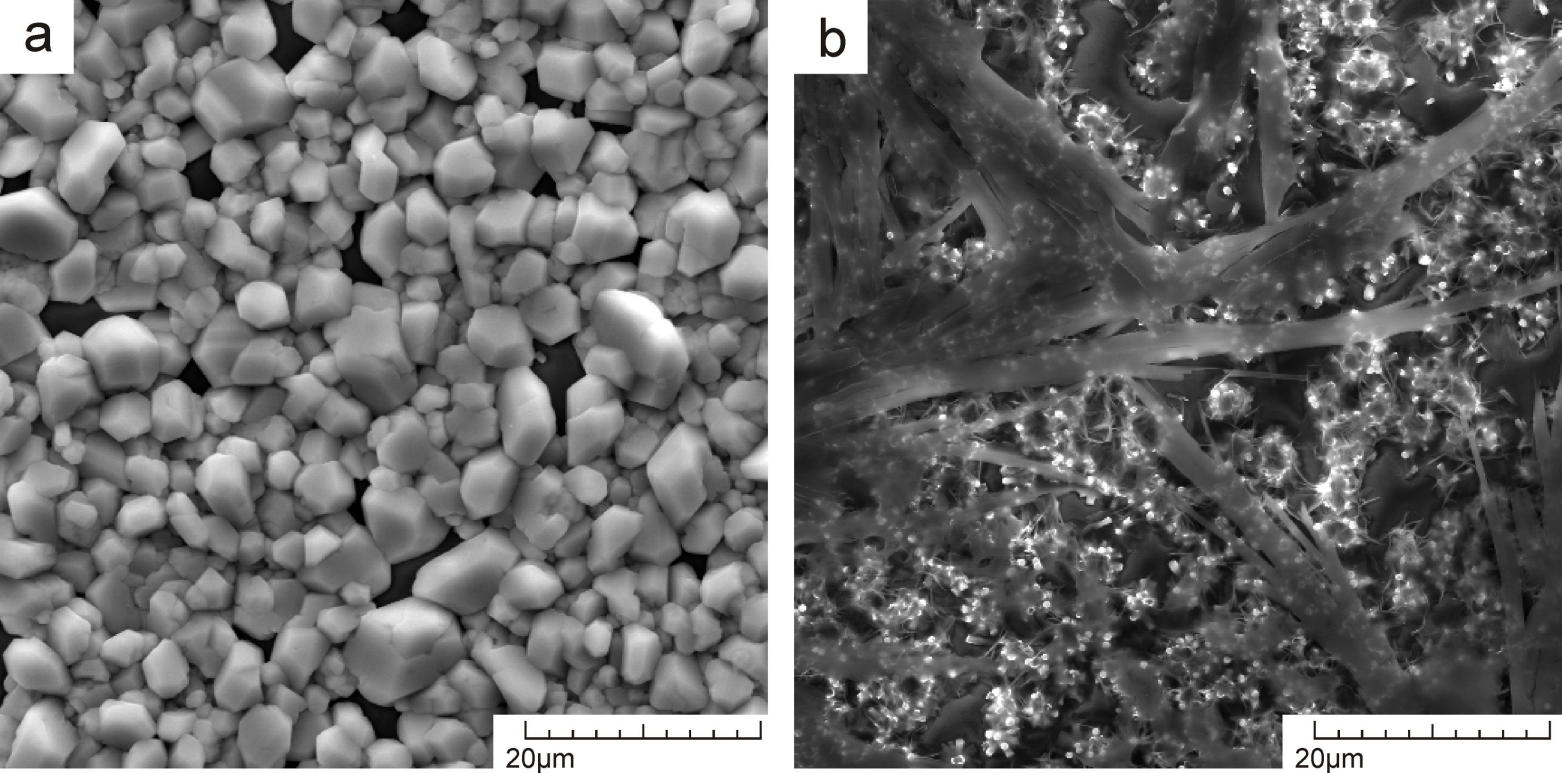
Sedimentation of (a) Pb(NO_3_)_2_ and (b) Cd(NO_3_)_2_ on the surface of ZnO nanotubes.

As can be seen from Figure 6, for the same solution concentrations, Pb(NO_3_)_2_ forms well-developed crystallites of lead oxides mixture, whereas Cd(NO_3_)_2_ forms a very thin amorphous layer on the ZnO surface. The SEM images were recorded using secondary electrons, whose output depth is within the range of 1–10 nm; the fact that the contours of the ZnO nanotubes (bright points) are easily visible through the film therefore indicates that the thickness of the Cd film does not exceed 10 nm. It can be concluded that Cd sediments form a very thin layer of horizontally oriented crystallites. This confirms that bonds between Cd ions and the ZnO surface are much weaker than between cadmium ions that prevent the rough ZnO surface to act as crystallization precursors in the heteroepitaxial growth process. In other words, particles will, with more probability, join an existing layer and increase its thickness rather than participate in the formation of a new precursor or the next layer.

The CV signal dependence on ZnO morphology can be explained as follows. There are two types of adsorption centres on ZnO nanostructure surfaces: on nonpolar lateral surfaces (such as the 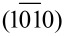) plane and its equivalent planes), and on polar surfaces that are largely intrinsic to structures with porous geometry and are associated with defects on metastable surfaces or on porous surfaces. The improvement of nanoneedle adsorption rates compared to nanorods can be attributed to an increase in the total lateral surface area, and as a result, an increase in the number of adsorption bonds [24–25]. Significant improvements in nanotube adsorption rates compared to similar diameter nanorods and nanoneedles are associated with both the increase in surface area (via the formation of a cavity) and the adsorption bonds due to surface defect sites. A large number of defects can be explained by the fact that, unlike nanorods and nanoneedles, which are formed in the hydrothermal growth process, nanotubes are formed in the etching process. Because of the remarkable deficiency of Zn ions, the aging process further suppresses the growth processes, and the probability of a new plane formation at the polar metastable plane (0002) is smaller than the probability of etching this plane by elements remaining in solution.

The role of adsorption bonds on surface defects and polar surfaces was tested using an additional morphology of adsorbent: ZnO nanoplates. This morphology was obtained by replacing HMTA with urea during the growth process, which resulted in inhibiting the growth of rods in the axial (c-axis) direction and stimulated growth in the lateral directions. As a result, the total surface area of (0002) planes increased, but the total surface area of lateral surfaces decreased.

**Figure 7:**
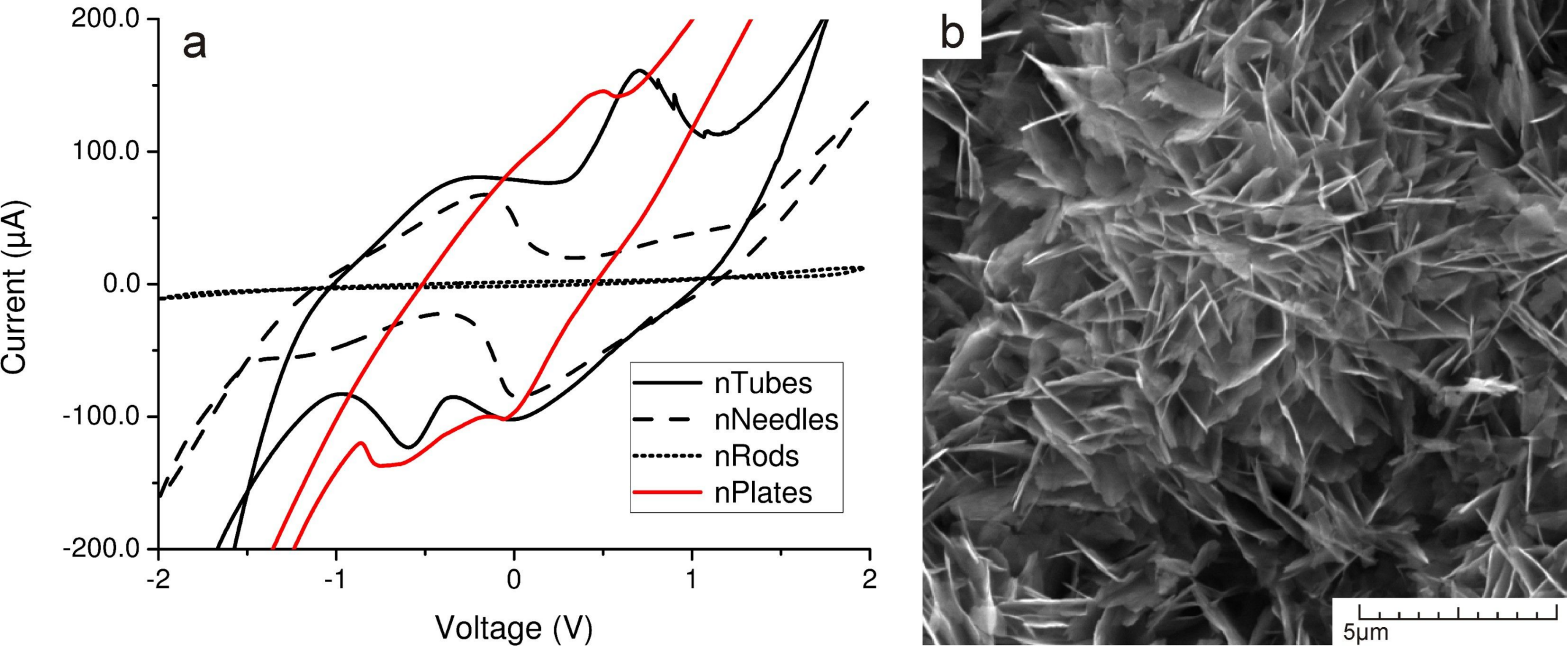
(a) CV curve for the concentration of 1.5 mM Pb(NO_3_)_2_ on ZnO nanoplates (red) compared to the previously used ZnO morphologies (black curves); (b) SEM image of as-synthesized ZnO nanoplates.

Figure 7 confirms that, in the case of ZnO nanoplates, the measured CV curve shows even better results in the reduction component than in the case of nanotubes, thereby proving the effectiveness of porous structures: adsorption bonds, which are located on surface defect sites or on metastable polar planes, have a better ability to form compounds with adsorbate than bonds, which are located on nonpolar surfaces.

It follows from the experiments that, in concentrated solutions, the CV measurements clearly reflect the process quality, whereas in the low concentration range, the measurements show poor sensitivity.

### Differential pulse voltammetry studies

In various papers, the DPV method was shown to be more sensitive and selective when compared to the CV method [28–30]. Therefore, the experiment was repeated with this method for all morphologies of the ZnO nanostructures. As an analyte, an aqueous solution of Pb(NO_3_)_2_ was used. The maximum concentration was chosen 10 and 100 times lower than CV sensitivity threshold of 1.5–3 mM.

**Figure 8:**
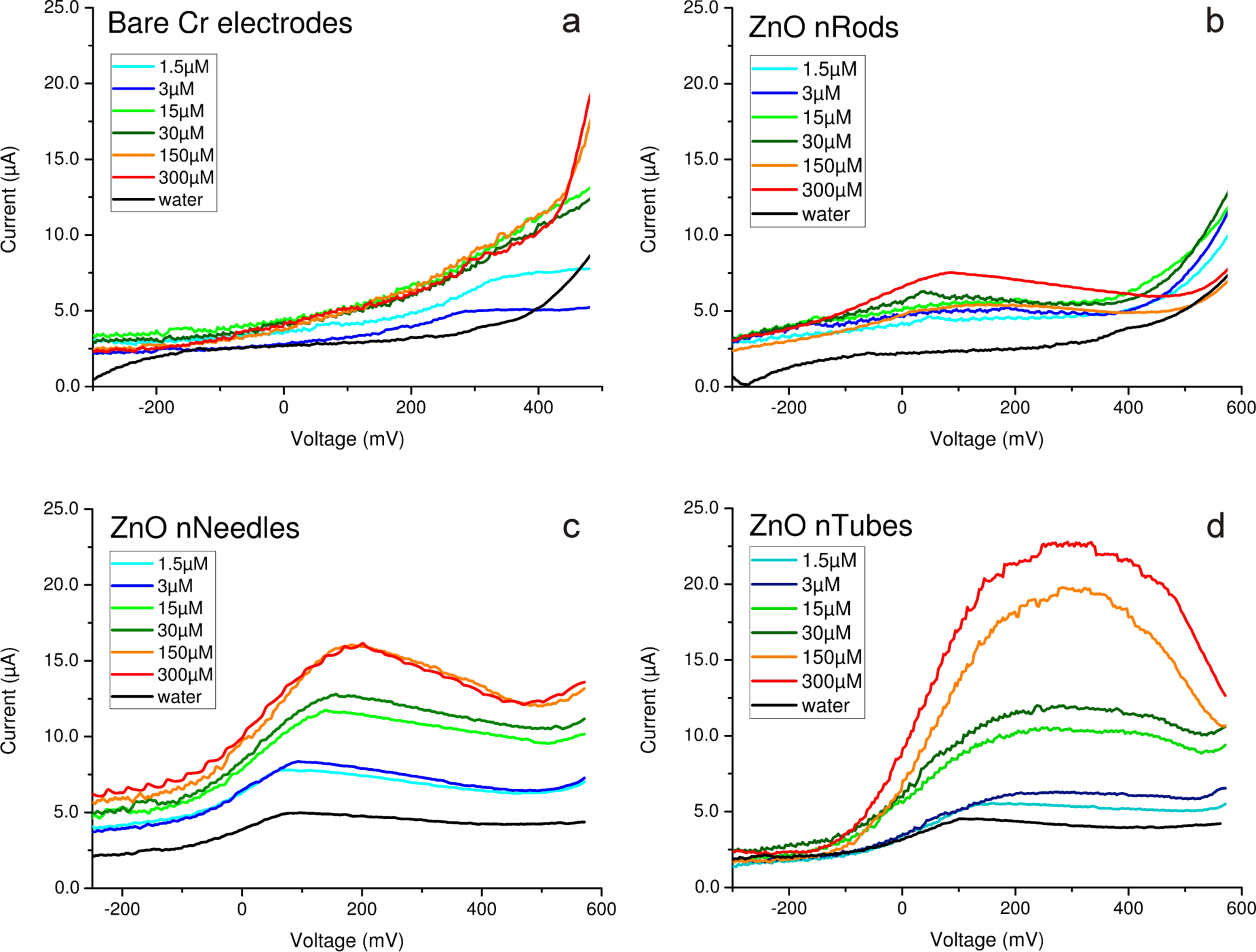
DPV curves for different ZnO morphologies: (a) untreated Cr electrodes, (b) nanorods, (c) nanoneedles and (d) nanotubes. As an analyte, a solution of Pb(NO_3_)_2_ at concentration of 1.5 µM to 300 µM was used.

As shown in Figure 8, the DPV method is actually more sensitive than the CV method: using the same electrodes, it is possible to detect concentrations that are 100 times smaller than the CV sensitivity threshold.

With regards to the role of morphology in ZnO nanostructures on sensitivity, the DPV measurements presented the same trend as the CV measurements: nanotubes, followed by nanoneedles, show the best results; the lowest sensitivity was observed on electrodes coated by nanorods. The current peaks did not appear on the untreated Cr electrodes; furthermore, the signal dependence on Pb(NO_3_)_2_ concentration was not observed. In the case of nanostructured electrodes, there is a strong dependence of the signal on analyte concentration (Figure 8): concentration duplexes were observed not only in the case of nanotubes, as was observed in CV measurements (Figure 4), but also in the cases of nanoneedles and even for nanorods.

## Conclusion

Overall, it can be concluded that the CV technique is suitable for qualitative process monitoring, but the DPV measurements are more suitable for quantitative analysis, where it is necessary not only to determine the presence of analyte in solution, but also the accurate concentration of analyte. The DPV technique also showed better sensitivity in the case of low analyte concentrations: it allows for the detection of concentrations of Pb(NO_3_)_2_ 100 times lower than the CV technique.

The effects of ZnO nanostructure morphology on the sensor sensitivity were also investigated. There is a clear tendency towards enhancing the intensity of the measured signal by increasing the surface area of working electrodes. In all measurements, nanotubes exhibited significantly higher sensitivity than nanorods and nanoneedles. This confirms that adsorption bonds associated with defect states contribute more to the process than the adsorption bonds, which are located on the nonpolar, side surfaces. This fact was also confirmed by an additional experiment on ZnO nanoplates.

The effectiveness of sedimentation is directly related to the electronegativity of the adsorbate: substances with higher electronegativity form stronger bonds with the ZnO surface and exceed the monolayer range. This was supported by the fact that a significantly better adsorption of lead ions was observed compared to cadmium ions on the surface of ZnO nanostructures. This tendency was observed for all morphologies across all concentrations of analyte.

In general, it can be concluded that ZnO nanostructures with porous morphology can be used as an effective heavy metal ion sensor on the one hand, and an effective adsorbent of these ions on the other.
